# Neuroprotective effect of heme oxygenase-2 knockout in the blood injection model of intracerebral hemorrhage

**DOI:** 10.1186/1756-0500-7-561

**Published:** 2014-08-22

**Authors:** Jing Chen-Roetling, Ying Cai, Raymond F Regan

**Affiliations:** Department of Emergency Medicine, Thomas Jefferson University, 1025 Walnut Street, College Building Room 813, Philadelphia, PA 19107 USA

## Abstract

**Background:**

The toxicity of heme breakdown products may contribute to the pathogenesis of intracerebral hemorrhage (ICH). Heme catabolism is catalyzed by the heme oxygenase enzymes. We have previously reported that heme oxygenase-2 (HO-2), the constitutive isoform, protects neurons from hemin in vitro and reduces oxidative stress after striatal blood injection. In order to further evaluate HO-2 as a therapeutic target, we tested the hypothesis that HO-2 gene deletion protects neurons and attenuates behavioral deficits after ICH.

**Findings:**

Injection of 20 μl blood into the right striatum of HO-2 wild-type mice resulted in loss of approximately one third of striatal neurons 4-8 days later. Neuronal survival was significantly increased in HO-2 knockout mice at both time points. This was associated with reduced motor deficit as detected by the corner test; however, no differences were detected in spontaneous activity or the adhesive removal or elevated body swing tests.

**Conclusion:**

HO-2 knockout attenuates perihematomal neuron loss in the blood injection ICH model, but has a weak and variable effect on neurological outcome.

## Findings

### Background

The heme oxygenase (HO) enzymes catalyze breakdown of heme to biliverdin, carbon monoxide, and ferrous iron. Parenchymal heme is present in abundance after intracerebral hemorrhage (ICH), and so the effect of these enzymes has been the subject of considerable research interest. The unavailability of selective and specific HO inhibitors has necessitated the use of knockout mice for these studies. Wang et al. reported that mice lacking HO-1, the inducible isoform, sustained less perihematomal injury after collagenase-induced ICH. This was associated with a reduction in the inflammatory response, but only a transient improvement in neurological outcome [1]. We have previously reported that HO-2 knockout protected murine neurons in vitro from hemoglobin or hemin [2, 3], and attenuated oxidative injury markers after hemoglobin or blood injection into the striatum [4, 5]. However, when ICH was produced by collagenase injection, HO-2 knockout either increased local cell injury or had no effect, while increasing neurological deficits to a variable extent [6, 7].

The animal model that is most representative of clinical ICH is undefined and somewhat controversial, necessitating evaluation of pharmacologic and genetic interventions in both models [8]. The negative effect of HO-2 knockout in the collagenase model indicated the need for further assessment of its impact in the blood injection model. In the present experiments, we used a recently-validated fluorescent method to quantify neuron loss in HO-2 wild-type and knockout mice after striatal injection of autologous blood. We also evaluated the effect of HO-2 knockout on neurological outcome, which had never been reported in this model.

### Methods

#### a) Experimental animals

All breeding and procedures were conducted following protocols approved by the Thomas Jefferson University Institutional Animal Care and Use Committee. Mice used in this study were bred in our animal care facility and expressed the red fluorescent protein variant dTomato in neurons [9]; recent experiments demonstrated that loss of dTomato fluorescence correlated well with neuron loss adjacent to a striatal hematoma [6]. Mice were crossed with homozygous HO-2 knockout mice (C57BL/6 x129/Sv background); heterozygous HO-2 knockout mice were then bred to obtain HO-2 WT and knockout mice. Prior experiments had demonstrated that neuronal dTomato expression had no effect on behavioral phenotype in either WT or HO-2 KO mice [6].

#### b) Striatal blood injection

Mice (n = 34, 18 males, 16 females) were 4-6 months old when used. Striatal blood injection (20 μl total volume) was conducted as previously described under 1.5% isoflurane anesthesia [10]. Temperature was monitored with a rectal probe and maintained at 37.0 ± 0.5 degrees with a heating lamp.

#### c) Striatal injury quantification

Striatal neuron loss was assessed 4 and 8 days after blood injection by dTomato fluorescence assay [6]. Striatal cell viability was quantified on the same samples via MTT assay [4, 5]. Both measures correlate well with more laborious counts of striatal neurons on histological sections using stereological methods [6]. After cervical dislocation under deep isoflurane anesthesia, brains were rapidly removed; injected and contralateral striata were immediately excised and dissociated separately by trituration. The cell suspension was incubated with 0.25 mg/ml MTT at 37°C for four minutes. After collection by low speed centrifugation, the formazan product was extracted in 2 ml isopropanol, and absorbance of this solution was determined (562 nm). Cells were again collected by centrifugation and sonicated in 500 μl PBS to release dTomato. Fluorescence (ex 557 nm, em 585 nm) was then quantified. Absorbance and fluorescence values were normalized to those in contralateral striata.

#### d) Behavioral testing

Neurological deficits were assessed by a blinded observer as previously detailed [6], via: 1) Digital analysis of spontaneous activity in a 3 hour video recording by HomeCageScan (Version 3.0, Clever Systems Inc., Reston, VA USA); 2) Corner test, right striatal injury produces a right turning bias [11]; 3) Adhesive removal test [12]; 4) Elevated body swing test, right striatal injury produces a left swinging bias [13].

#### e) Statistical analysis

Differences between wild-type and knockout groups were assessed via unpaired t-test using GraphPad software.

### Results

### a) *HO-2 knockout reduces perihematomal injury*

**Figure 1:**
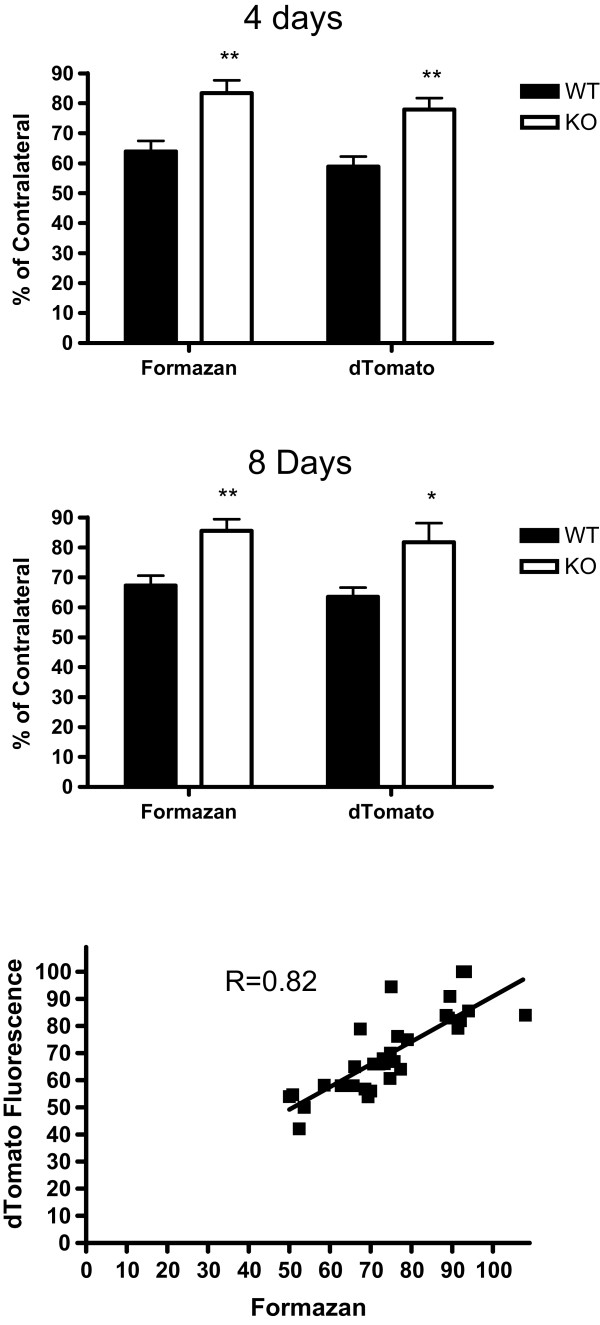
**Right striatal cell injury 4 and 8 days after blood injection, quantified by MTT assay (Formazan) and extracted dTomato fluorescence, in HO-2 wild-type and KO mice.** All values (mean ± S.E.M, n = 7-9/condition) are normalized to those in the contralateral striatum (=100). *P < 0.05, **P < 0.01 v. corresponding WT condition. Scatter plot demonstrates correlation between MTT and dTomato assay results.

In HO-2 WT mice, striatal dTomato fluorescence at 4 and 8 days after blood injection was 58.8 ± 3.4% and 63.5 ± 3.1% of contralateral, respectively (Figure 1). In HO-2 KO mice, this value was significantly increased at both time points. Consistent with prior observations [6], the percentage change in dTomato expression in injected striata was in close agreement with the decrease in MTT conversion to formazan.

#### b) Effect of HO-2 knockout on behavioral recovery

**Figure 2:**
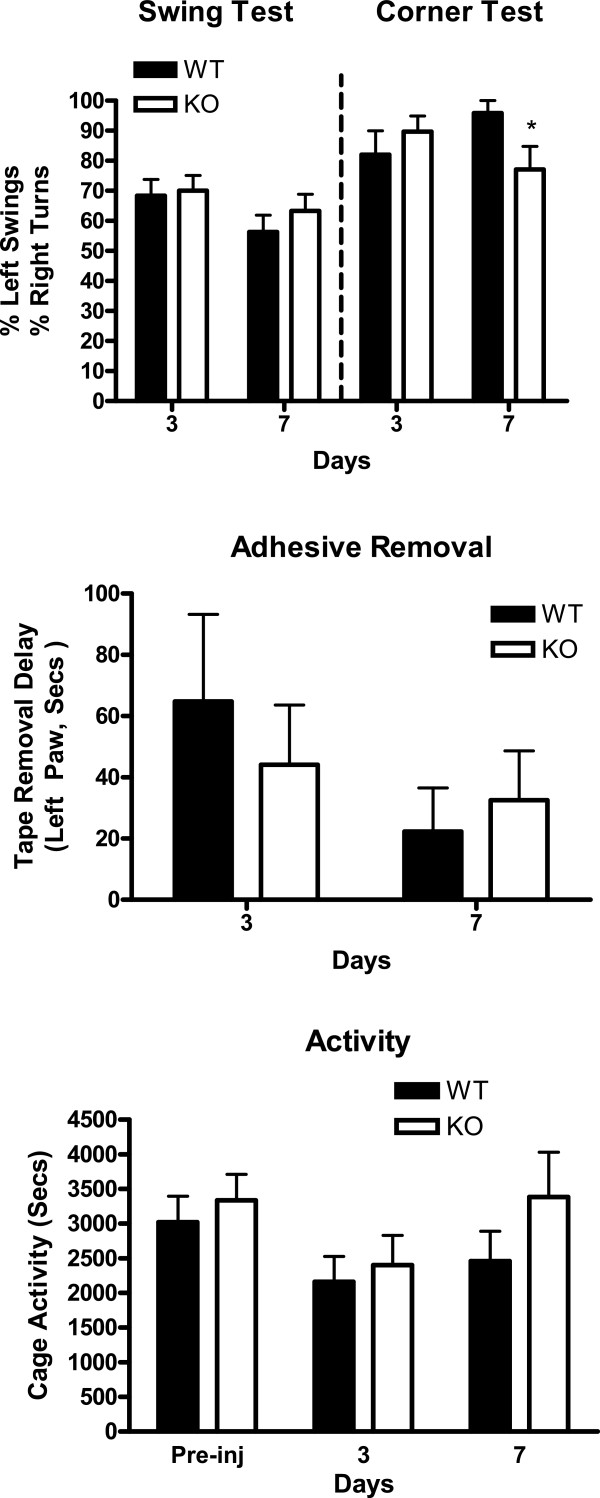
**Effect of HO-2 knockout on behavioral deficits in striatal blood injection model.** Sensorimotor deficits were assessed by corner test (right striatal injury causes turn to right), elevated body swing test (injury causes left swing), adhesive removal test, and digital analysis of spontaneous activity, n = 8-13/condition, *P < 0.05 v. corresponding WT condition.

Injection of blood into the right striatum produced focal left sided deficits and a reduction in spontaneous cage activity at Day 3, with partial recovery noted by Day 7 (Figure 2). A significant difference between HO-2 WT and KO groups was observed only at day 7 in the corner test. HO-2 KO did not have a significant effect on recovery of spontaneous cage activity, or on focal sensorimotor deficits as detected by adhesive removal or elevated body swing tests.

### Discussion

This study serves two purposes. First, we have demonstrated the neuroprotective effect of HO-2 knockout in this ICH model, using a novel fluorescent method to quantify perihematomal neuron loss. Second, we present data indicating that this cytoprotection was associated with a rather weak and variable improvement in behavioral outcome.

Accurate assessment of cell death in tissue surrounding an experimental hematoma is complicated by the heterogeneity of the injury. In contrast to the infarction produced in ischemic stroke models, an experimental hematoma is surrounded by areas of variable neurodegeneration [14]. This presents considerable challenges to section and field selection when cell loss is quantified by traditional histological methods. Accurate and unbiased results can be obtained with design-based stereology, but this approach is expensive, laborious, and rarely employed in ICH studies. Using the collagenase ICH model, we have recently reported that striatal neuron loss can be accurately estimated in mice expressing neuronal dTomato by simply measuring the fluorescence of a tissue lysate [6]. The present study uses this approach for the first time in the blood injection ICH model. Striatal fluorescence measurements correlated closely with cell viability as measured with the MTT assay, a method that has previously been validated with stereological cell counts [6].

The neuroprotective effect of HO-2 knockout in this model is consistent with our prior findings that it attenuated protein and lipid oxidation after striatal hemoglobin or blood injection [4, 5]. The disparate effect of HO-2 knockout in the collagenase and blood injection models likely reflects the predominance of differing injury mechanisms in these models. When equal hematoma volumes were compared, a collagenase-induced ICH produced significantly more blood-brain barrier injury and edema [15]. Disruption of multiple vessels near the injection site may also produce ischemia, which is not a feature of the blood injection ICH model [16] and has not been observed in blood flow studies of patients sustaining an ICH [17]. It is possible that any benefit of HO-2 knockout on the heme-mediated component of injury after collagenase-induced ICH is negated by its deleterious effect on ischemic injury, as previously reported by Doré et al. [18].

Since neurological outcome will be the key measure of efficacy in any clinical trial, identification of a therapeutic target for ICH requires clear and convincing demonstration of functional improvement in experimental models. To date, that standard has not been met for either HO isoform. Studies by Wang and Doré demonstrated that HO-1 KO improved the neurologic score at 24 hours after collagenase injection but not at later time points, suggesting a limited utility of targeting HO-1 alone [1]. HO-2 knockout has had variable but negative effects on behavioral outcome after collagenase-induced ICH [6, 7], and a weak benefit in the present study. Concomitant inhibition of both HO-1 and HO-2 with porphyrin HO inhibitors reduced edema and neuronal loss after experimental ICH, and may be more effective than targeting a single isoform [19–21]. Although the nonspecificity of these compounds limits their value as mechanistic probes, further investigation of their effects on neurological outcome in both ICH models seems warranted.

### Conclusion

HO-2 knockout attenuates perihematomal neuron loss in this murine blood injection ICH model, but has a weak and variable effect on neurological outcome. These results do not support the utility of therapies that target HO-2 activity or expression per se after ICH.
